# Antioxidant and nephro-protective effect of *Juglans regia* vegetable oil against lead-induced nephrotoxicity in rats and its characterization by GC-MS

**DOI:** 10.17179/excli2018-1235

**Published:** 2018-05-23

**Authors:** Ahlem Soussi, Manel Gargouri, Amel Akrouti, Abdelfattah El Feki

**Affiliations:** 1 Animal Eco-physiology Laboratory, Faculty of Sciences, University of Sfax, Tunisia

**Keywords:** antioxidant activities, GC-MS, Juglans regia, nephrotoxicity, lead, rats

## Abstract

Lead (Pb) intoxication remains a major health hazard causing various deleterious effects especially on renal and hematologic system. The current study elucidated the potential protective effect of JRVO against nephrotoxicity induced by lead. Male rats were randomly divided into three groups: group one (control) received ad libitum distilled water and 1 mL of saline solution (0.9 %) given by intra-peritoneal (i.p) injection, group two (Pb) was kept on tap distilled water and animals were i.p, injected daily with lead every two days from day five until day ten, namely the sacrifice day, and group three (Pb+J) was administered by intra-peritoneal injection of Pb with the same dose and same way with Group two, while JRVO extract was administered daily by gavage during ten days. The exposure of lead reduced the number of red and white blood cells. Besides, plasma biomarkers (urea, uric acid, creatinine, LDH and ALP) levels were reduced. Lipid and protein per-oxidations increased and objectified by high TBARS and PCOs levels, while glutathione peroxidase, superoxide dismutase and catalase activities showed a significant decline after ten-day treatment. Conversely, the JRVO prevented kidney biomarker changes by improving hepatotoxicity induced by lead as evidenced by restoring the biochemical markers cited above to near normal levels. Kidney histoarchitecture confirmed the biochemical parameters and the beneficial role of JRVO. It can be concluded that the administration of JRVO alleviates Pb-induced toxicity, thus demonstrating its potent antioxidant efficacy.

## Introduction

For many years, lead has been recognized as a toxic metal that induces a wide range of behavioral, biochemical and physiological effects on humans, animals as well as soils and sediments through aerial waste deposited by metal industries. Gasoline in cars and shotgun pellets constitute other main sources of lead (Rainio et al., 2015[43]). Lead does not have any beneficial effects on humans, and its presence at high concentrations produces very undesirable toxic consequences to humans affecting all the body organs (Ibrahim et al., 2012[31]; Markowitz, 2011[37]) Once absorbed, the Pb is bound to erythrocyte proteins and will accumulate in soft tissues and bone (Garcia-Nino and Pedraza-Chaverri, 2014[21]). In fact, the liberation of reactive oxygen species (ROS) such as superoxide radicals, hydrogen peroxide, hydroxyl radicals and lipid peroxides is potentially generated by lead which is an essential factor of oxidative stress. Its toxicity resulted in reducing the levels of antioxidants in the blood as well as certain enzymes like catalase and superoxide dismutase (Bennet et al., 2007[9]; Singh et al., 2013[54]). Also, such toxicity ended in an elevation in malondialdehyde (MDA) levels (Dogru et al., 2008[16]; Sharma et al., 2014[52]). Lead-induced toxicity, related to increased oxidative stress, derives from an imbalance between the production of reactive oxygen species (ROS) and antioxidant capacity (ATSDR, 2007[7]). However, the kidneys, which are the main organs of the body maintaining the internal environment, are susceptible to be damaged by drugs and environmental chemicals (Apaydın et al., 2016[6]). In the kidneys, lead intoxication may cause proximal tubular dysfunction or irreversible nephropathy depending on the exposure regimens (Singh et al., 2013[54]; Guimarães et al., 2012[25]).

Some oxidative-related disorders and carcinogenic events in humans can be prevented or treated by the intake of antioxidant compounds, compounds that are able to ameliorate or enhance the biological antioxidant mechanisms (Havsteen, 2002[28]). To overcome oxidative stress, the use of medicinal and phenolic plants, which have an antioxidant activity for protection against heavy metal toxicity, has become a point of interest for phototherapy researchers (Senapati et al., 2001[51]; Hsu et al., 1997[30]).

*Juglans regia* Vegetable oil (JRVO) enjoys the reputation of being a panacea because of its wide range of medicinal effects. It has been recognized for its antioxidant properties and its positive effects against oxidative stress. JRVO has been continuously used to treat diverse ailments including diarrhea, hyperglycemia, cancer, and any infectious disease among humans (Panth et al., 2016[40]). Previous studies, demonstrated that the beneficial health effects of *Juglans regia* oil had been mainly attributed to its richness in polyunsaturated fatty acids (more than 70 %). Juglans oil is excellent if used fresh for edible purposes because it is rich in the so-called ω-3 and ω-6 fatty acids that are essential for the human diet (Poggetti et al., 2018[41]).

As there is a lack of data regarding the interaction between Pb and JRVO, *in vivo*, the aim of the present study was to examine the potential protective effect of this oil on the lead nephrotoxicity in rats together with its relationship with oxidative stress. 

## Methods and Materials

### Chemicals reagents

Lead acetate Pb (C_2_H_3_O_2_)_2_ was obtained from SD Fine Chemicals. However, *Juglans regia *oil was gained from “Laboratoire Altho” (Monfort, France), thus used in all experiments. All other chemicals and products were used in the study and purchased from Sigma-Aldrich (Sigma-Aldrich, Sfax, Tunisia).

### Animal's treatments and experimental design

Thirty-six adult male Wister rats, aged 11-12 weeks and weighing 200 g, were used. The animals were housed in cages under controlled laboratory conditions (21±1 °C, 10 h/ 14 h light/dark cycle and 40±10 % of humidity). Food and water were provided with ad libitum. 

After one week of acclimatization, the animals were randomly divided into three groups of six rats as follows:

- Group One (Controls): rats received distilled water injected intarperitoneally 1 mL of saline solution (0.9 %).
- Group Two (Pb): rats received distilled water injected daily with lead (0.344 g/kg body weighs) (Gargouri et al., 2016[23]).
- Group Three (Pb+J): rats received oral gavage with JRVO (0.9 g/kg body weight) associated with lead (0.344 g/kg body weighs) injected intraperitoneally.

The treatments were monitored during 5 and 10 days (Soussi et al., 2006[56], 2017[55]). To avoid stress, animals from different groups were sacrificed by cervical decapitation. Plasma was separated and recovered, in heparinized tubes, by centrifugation at 3000 × g 15 min^−1^. Samples of the kidney were excised, accurately weighed, fixed in Bouin's solution and processed for histology. Briefly, the fixed tissues were embedded in paraffin, cut to produce 4-5 μm sections and stained with hematoxylin and eosin for examination with light microscope. Kidney tissue was minced, rinsed, homogenized in Phosphate buffer solution (TBS, pH 7.4) and centrifuged at 9000 x g for 20 min. The resulting supernatants were stored at -80 °C for various biochemical assays. All samples were analyzed in triplicate.

### Oil analysis

Fatty acid composition was identified by Gas chromatography-mass spectrometry (GC-MS) methodology using an Agilent 6890N Network GC system (Agilent Technologies). The chromatograph gas was fitted with a splitless injector for a low background DB-5MS fused silica capillary column (50 m × 0.25 mm i.d. × 0.25 µm film in thickness). The oven temperature was initially held at 175 °C for 13.5 min, then increased to 185 °C at a heating rate of 2 °C/min up and held isothermal for three minutes. Injector and FID detector temperature were held at 220 and 280 °C. The identification of compounds was carried out by Chem-Station software package (Agilent Technologies). FAMEs were identified by comparison of their retention time with regard to pure standards and were quantified according to their percentage area in the lipid fraction.

### In vitro assays

#### DPPH-scavenging activity

The hydrogen donation of the extract was measured using the DPPH method described by Hatano et al. (1988[27]). 250 μL of DPPH radical solution (0.2 mM) was added to each test. All test tubes were incubated for 30 minutes in the dark. The absorbance of the resulting solutions was measured at 517 nm. The IC_50_ was calculated using the following formula:

IC_50_ (%) = [(A_0_−A_1)_/A_0_]*100 (1)

Where A_0_ and A_1_ are the optical densities of the control and the sample at 30 min, respectively. BHT was used as a positive standard and all samples were analyzed in triplicate.

#### Hydrogen peroxide radical scavenging activity

The abilities of JRVO to scavenge H_2_O_2_ were determined according to the method of Ruch et al. (1989[49]). A stock of aqueous solution of *Juglans regia* oil (1 mg/ml) was prepared. Different amounts of the stock solution were transferred into different test tubes and different amounts of the stock solution were transferred into different test tubes. 0.6 ml of hydrogen peroxide solution (40 mM) was prepared in phosphate buffer saline (0.1 M; pH 7.4) and added. Then, the reaction mixtures were incubated for 10 minutes. Absorbance of mixture at 230 nm was determined against a blank solution prepared in the same way without a sample using UV-Visible spectrophotometer.

The percentage scavenging of hydrogen peroxide of *Juglans regia* and gallic acid at different concentrations was calculated using this formula:

*% Scavenging H_2_O_2_ = [1- (Absorbance of extract/Absorbance of control)] x100 *(2)

#### Ferric Reducing Antioxidant Power (FRAP) or FRAP assay

The capacity of the extracts to transform Fe^3+^ to Fe^2+^ was according to the method of Megías et al. (2009[39]). The absorbance was read at 700 nm. BHT was used as an authentic standard. The IC_50_ values were calculated from linear regression analysis. 

### In vivo assays

#### Hematological analysis

Red and white blood cells were counted in blood samples after dilution in the Hayem and Turck solutions respectively. Blood cells count was performed, by transferring blood sample onto counting lamella, and examined under a light microscope with a magnification of 400 (Blaxhall and Daisley, 1973[11]; Blaxhall, 1981[10]; De Wilde MA and Houston, 1961[15]). Hematocrit was determined according to Wilhelm Filho et al. (1992[57]) and Jewet et al. (1991[32]) method.

#### Plasma analysis

Plasmatic urea, uric acid and creatinine levels were estimated using commercial diagnostic kits, (ref 20152, 20142, 20092, Biomaghreb). Creatinine Clearance was calculated using the formula (Charrel, 1991[13]): 

Creatinine clearance = U.V/P

Where U, V and P are the urinary creatinine level, the volume of urine sample was collected within 24 h and the plasma creatinine concentration respectively.

#### Protein quantification

Kidney protein contents were measured according to Lowry et al. using bovine serum albumin as standard (Lowry et al., 1951[36]).

#### TBARS and PCO assays in kidney

The level of lipid peroxide in kidney was determined spectrophotometrically according to Yagi (1976[58]). The development of pink color with the absorption at 530 nm and the TBARS values were calculated and expressed in nmol/mg protein.

Protein carbonyl (PCO) content in kidney tissue was determined using the method by Reznick and Packer (1994[46]). The absorbance using a spectrophotometer was measured at 370 nm. The carbonyl content was calculated, in nmol/mg protein, using the molar extinction coefficient of DNPH (2,4-Dinitrophenylhydrazine) (ɛ = 2.2 x 104 cm/M).

#### Enzyme assays

SOD activity was determined according to the assay of Beauchamp and Fridovich (1971[8]) based on the photo reduction of nitroblue terazolium (NBT). The absorbance was recorded at 560 nm and the activity was expressed as units / mg protein.

CAT was assayed by the decomposition of hydrogen peroxide according to Aebi (1984[2]) method. Decrease in absorbance due to H_2_O_2_ degradation was monitored at 240 nm and the enzyme activity was expressed as µmol H_2_O_2_ consumed/min/mg protein.

The GPX activity was determined in the kidney cytosolic fraction according to Flohe and Gunzler (1984[19]). The enzyme activity was expressed in nmoles of GSH oxidized/min/ mg protein.

#### Histopathological examination

All histopathology examinations were performed using standard laboratory procedures. Kidneys were embedded in paraffin, sectioned at a thickness of 5 µm, and stained with haematoxylin-eosin (Gabe, 1968[20]). Six slides were prepared from each kidney tissue and all sections were evaluated for determination of the tubular and glomerular injuries' degree.

### Data analysis and statistics

Statistical analysis was performed using one-way analysis of variance (ANOVA) followed by Fisher's test. All values were expressed as means ± SE. Differences were considered significant, set at p < 0.05.

## Results

### In vitro assays

#### Analytical parameters of JRVO and its compounds

JRVO analytical parameters (fatty acid) are presented in Table 1(Tab. 1). JRVO contained 7.69 % of saturate (palmitic and stearic acids) and 91.65 of monounsaturate (mainly linoleic acid).

#### DPPH radical scavenging assay

The antioxidant activity of JRVO was measured by the ability to scavenge DPPH free radicals. It was compared with the standard antioxidant, ascorbic acid. The JRVO antioxidant activity was measured at 517 nm. Indeed, a lower absorbance indicated a higher radical scavenging activity of the oil compared with the standard antioxidant which is ascorbic acid. The IC_50_ values were recorded as 0.2 mg/ml for JRVO and 0.15 mg/ml for ascorbic acid. *Juglans regia *oil represents a concentration dependent on decrease in absorbance which is shown in Table 2(Tab. 2).

#### H_2_O_2_ radical scavenging assay

The radical scavenging property of a compound, which may serve as a significant indicator of its potential antioxidant activity, was determined using H_2_O_2_ radical scavenging assay. The scavenging ability of JRVO on H_2_O_2_ is shown in Table 2(Tab. 2) and compared with gallic acid as control standard. *Juglans regia* oil and gallic acid demonstrated hydrogen peroxide radical scavenging activity in a dose-ependent manner. The H_2_O_2_ radical scavenging increased from 36.99 ± 0.66 % to 56.52 ± 0.5 % when the concentration of the oil increased from 0.25 to 2.5 mg/ml. The IC_50_ value of VJRO and gallic acid were 2 mg/ml and 1.75 mg/ml, respectively (Table 2(Tab. 2)).

#### FRAP assay

Table 2(Tab. 2) illustrates the reductive ability of JRVO. Thus, *Juglans regia* oil has a potential to reduce most Fe^3+^ ions similar to the ascorbic acid. Both demonstrated the same IC_50_ values.

### In vivo assays

#### Body, absolute and relative kidney weights

During all experimentation period, Pb treatment induced a significant decrease in the absolute kidney weight and the Organo-Somatic Index as compared with control rats (p<0.001). The absence of mortality, in all treated groups, was also noticed. When JRVO was supplemented to Pb treated rats, a recovery occurred in absolute and relative kidney weights (Table 3(Tab. 3)).

#### Hematological parameters

Compared with the control group, red and white blood cells' number was reduced by 28.6 % and 16 % respectively in Pb-treated group. Other erythrocyte parameters such as mean globular volume and hematocrit were increased in this group by 16.4 % and 37.8 % respectively (Table 4(Tab. 4)). Co-treatment with JRVO improved all the parameters cited above to near normal values.

#### Biomarkers of kidney damage

In Pb treated rats, LDH activity, a biomarker of membrane kidney damage, was increased in plasma by 106 % when compared with those of controls. Conversely, the plasma levels of ALP were significantly lower in intoxicated (-99 %) rats than in controls. Table 5(Tab. 5) demonstrated that JRVO treatment ameliorated LDH and ALP activities when compared with those of lead treated rats.

Among other kidney specific biomarkers analyzed in our experiment, our results showed that creatinine, urea and uric acid and BUN levels were significantly higher in Pb-treated rats than in the controls. Supplementation of Pb-intoxicated rats with JRVO improved renal functions as manifested in the recovery of control levels of plasma markers (Table 5(Tab. 5)).

#### Estimation of TBARS, PCO and enzymatic antioxidant levels in kidney

Our results showed a significant increase in lipid peroxidation and proteins oxidation PCO by 90.4 % in Pb-treated rats when compared with those of controls. These modifications were significantly alleviated following co-administration of JRVO (Table 6(Tab. 6)).

Table 6(Tab. 6) depicts the activities of antioxidant enzymes (SOD, CAT and GPx) in the kidney of controls, Pb and Pb+J treated rats. A significant decrease of SOD (-50.8 %), CAT (-22 %) and GPx activities (-43.8 %) levels was shown in rats exposed to lead when compared with controls. Co-treatment of lead treated rats with JRVO improved enzymatic antioxidant activities.

#### Histopathological examination

**Figure 1:**
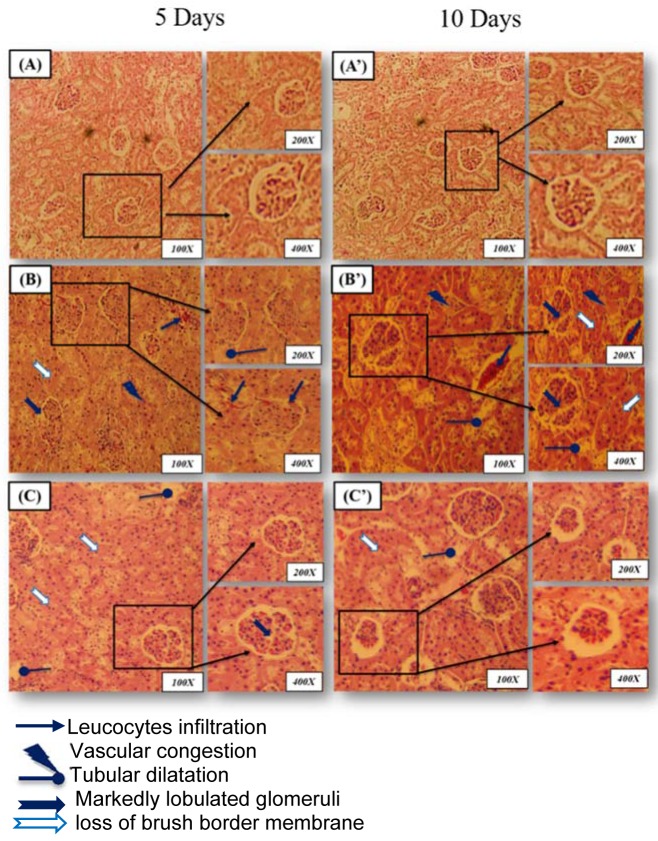
Microscopic observations of rat kidney were stained with hematoxylin-eosin (magnification: 100*x*, 200*x* and 400*x*) (A, A'): control group (C) showing normal glomeruli and renal tubules (distal and proximal) architecture after 5 and 10 days of treatment; (B, B'): Lead-treated group (Pb) showing a severe tubular destruction or dilatation after 5 days of treatment accompanied by glomeruli hypertrophy, loss of brush border membrane and vascular congestion after 10 days of treatment; (C, C'): Rat treated with *Juglans regia* oil and intoxicated with lead (Pb + J) showing marked improvement in the histological sections with normal glomeruli and tubules (distal and proximal) after 5 and 10 days of treatment, respectively.

Histological changes in the kidney of control and treated groups are depicted in Figure 1(A-A')(Fig. 1). Kidney of the control group showed normal histological structure with normal parenchyma and glomeruli. However, lead induced a severe tubular destruction or dilatation after 5 days of treatment accompanied by glomeruli hypertrophy, loss of brush border membrane and vascular congestion after 10 days of treatment (Figure 1(B-B')(Fig. 1)). This pathological aspect was alleviated by the administration of JRVO to P-treated group marked by an improvement in the histological sections with normal glomeruli and tubules (distal and proximal) after 5 and 10 days of treatment, respectively (Figure 1(C-C')(Fig. 1)). These histological studies were in agreement with the results of renal functional markers, the kidney lipid peroxidation and antioxidant status (Table 7(Tab. 7)).

## Discussion

Lead intoxication is one of the greatest hazards to human health that affects people at all ages. It is considered as one of the most toxic agents to various organs of the body as well as to metabolic pathways. Kidney, which is a major organ involved in the detoxification and metabolism of toxic substances, is consequently susceptible to the oxidative reaction of lead (Ponce-Canchihuamán et al., 2010[42]; Conterato et al., 2007[14]). In the kidney, lead intoxication may also cause proximal tubular dysfunction or irreversible nephropathy depending on exposure regimens (Ponce-Canchihuamán et al., 2010[42]; Conterato et al., 2007[14]; Adeyemi et al., 2009[1]).

The antioxidant effects on brain (Kuang et al., 1996[35]) and liver (Gyamfi et al., 1999[26]) were positively correlated with medicinal herbs supplementation. However, no study is made about the effects of *Juglans regia *vegetable oil on toxic agent-induced acute renal failure. Our investigation opted to evaluate the influence of JRVO against lead induced renal damage by assessing renal functional parameters and histopathological changes.

In the current study, the exposure of adult rats to lead during 10 days profoundly affected their hematological parameters. A reduction in red and white blood cells' number was observed. Other hematological parameters, namely globular volume and hematocrit, increased. Previous studies have shown that lead can induce hematological disturbances resulting from abnormalities in cell differentiation and hemoglobin synthesis during hematopoiesis (Rio et al., 2001[47]). Co-administration of JRVO improved the parameters cited above. This protective effect has not been reported earlier, and this is the first report on the hematological system. 

In the present investigation, a significant decrease in body and absolute kidney weights as well as OSI, was observed in the Pb treated rats. Similar results have been found by Gargouri et al. (2018[22]) in lead treated rats at a dose of 6 g/L lead acetate (via drinking water) for 21 days. The decline of body weight could be explained by a metabolic disorder causing energy metabolism pathways which interfere with lead. Weight loss can be linked also to excessive tissue protein breakdown (Andallu and Varadacharyulu, 2003[5]). In our experimental study, administration of JRVO to Pb-treated group improved body and kidney weights, which could be attributed to its high antioxidant capacity to exert a protective effect against oxidant-induced cell injury in renal cortical slices (Ahn et al., 2002[3]).

Changes of body and kidney weights in lead treated rats were associated with an increase of blood urea and serum creatinine which is considered as the marker of kidney impairment according to Karahan et al. (2005[34]). In the present study, an increase of plasma creatinine and urea reflected the diagnosis of renal failure (Donadio et al., 1997[17]). Similar results, reported by Yanardag and Sacan (2007[59]), demonstrated that elevated blood urea is known to be linked with an increased protein catabolism. This is due to the elevation of arginase enzyme production which is responsible for the hyperuricemia. Ruilope and Garcia-Puig (2007[50]) reported urea increase as a renal risk factor. Another biochemical marker, used in the current study to evaluate kidney function, was uric acid levels in plasma. It is known for its antioxidant capacity that presents the end product of purine catabolism based on its ability to reduce oxidative stress by scavenging various reactive oxygen species (Regoli and Winste, 1999[45]), peroxynitrite in particular (Hooper and al., 1998[29]). After exposure to lead, an increase of plasma uric acid level was observed. These findings corroborate previous studies elaborated in our laboratory (Gargouri et al., 2018[24]). The co-treatment of JRVO significantly attenuated the lead-induced kidney impairment indicated above. This protection is indicative of JRVO fatty acids and polyphenols richness (Poggetti et al., 2018[41]).

As previously described, kidney function impairment could be attributed to oxidative damage probably caused by lead (Gargouri et al., 2018[24]). Several lines of evidence have demonstrated that lead altered membrane integrity and fluidity followed by leakage of cellular enzymes into blood via an increased ROS generation (Amamou et al., 2015[4]; Celik and Suzek, 2008[12]). Our experimental data showed a state of oxidative stress in rats exposed to lead affirmed by the significant elevation in kidney TBARS, the index of lipid peroxidation. In addition, LDH, an intracellular enzyme, increased in the plasma of Pb treated group, confirming the increase in membrane permeability. Furthermore, Pb generated an important decrease of ALP values suggesting that markers of nephrotoxicity (ALP and LDH) were mainly affected by lead treatment (El-Tantawy, 2016[18]).

Free radicals generation during lead-treatment is correlated with deleterious effects on proteins, including PCO, an early marker for protein oxidation. In the present work, oxidative protein damage may be one of lead-induced oxidative stress and ROS stimulation (Kalender et al., 2015[33]), as demonstrated in the increase of PCO levels in kidney. Treatment with *Juglans regia* oil prevented oxidative damage induced by lead in the renal tissue due to its high content of polyphenols which are excellent antioxidants (Shimoda et al., 2008[53]). Our results were in agreement with previous reports of Ros et al. (2004[48]) and Zambón et al. (2000[60]) who demonstrated that juglans intake does not promote lipid peroxidation.

The decrease in the activities of SOD, CAT and GPx is indicative of enzymes' inhibition due to excessive ROS production induced by lead exposure in the treated rats. These results are in keeping with the previous reports (Gargouri et al. (2018[22]).

Conversely, the amelioration of markers' enzymes following co-treatment with JRVO owes to the presence of the nephroprotective agent such as fatty acids and polyphenols (Poggetti et al., 2018[41]). Similarly, Rather et al. (2012[44]) demonstrated that the JRVO exhibited remarkable antioxidant and scavenging activities.

The biochemical parameters were correlated with renal histological studies. The histopathological observation in lead-treated rats showed a severe tubular destruction or dilatation after 5 days of treatment accompanied by glomeruli hypertrophy, loss of brush border membrane and vascular congestion after 10 days of treatment. Our study also reports that JRVO marked improvement in the histological sections with normal glomeruli and tubules (distal and proximal) after 5 and 10 days of treatment, respectively. The beneficial health effects of Juglans oil have been mainly attributed to its high oleic (ω-9), linoleic (ω-6) and especially alpha linolenic (ω-3) acids' content. This fatty acid increased the expression of HO-1 in kidney which plays an important anti-apoptotic role following ureteral obstruction (Rather et al., 2012[44]).

## Conclusion

To encapsulate and based on the above findings, JRVO supplementation presents a protective antioxidant effect against lead intoxication resulting in oxidative damage, alteration of biochemical parameters and histopathology. Health beneficial effects of JRVO have been unambiguously documented and can partially explain the observed nephro-protective effect. The present study, therefore, provides biological evidence supporting the usefulness of *Juglans regia* vegetable oil against lead-induced toxic oxidative stress on the kidney tissue among a rat.

## Acknowledgements

The present work was supported by DGRST Grant (Direction Générale de la Recherche Scientifique et Technique-Tunisie (Appui à la Recherche Universitaire de base UR/13 ES-73).

The authors are grateful to Prof. N. Khedemi, ESP English teacher (Faculty of Sciences of Gafsa) for proof-reading the manuscript.

## Conflict of interest statement

The authors report no conflicts of interest associated with this manuscript.

## Figures and Tables

**Table 1:**
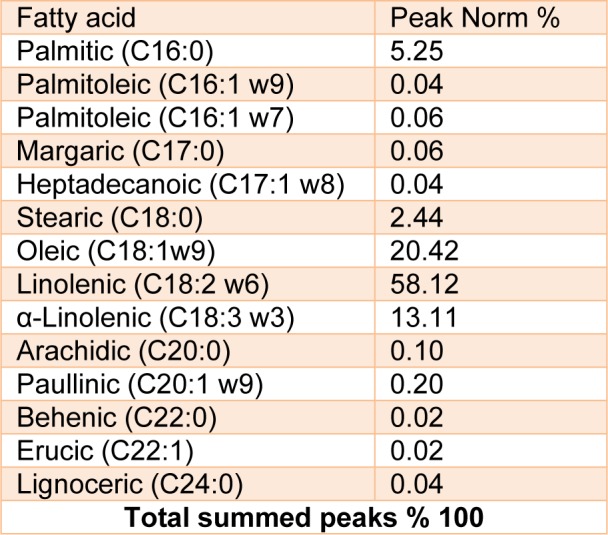
Fatty acid composition of *Juglans regia* oil

**Table 2:**
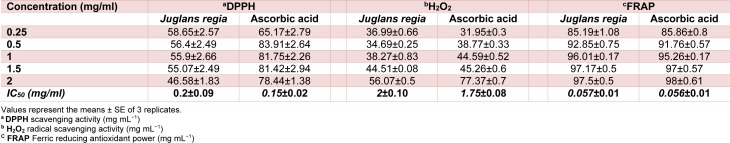
Antioxidant activities and phenolic contents in the ethanolic extract of *Juglans regia*

**Table 3:**
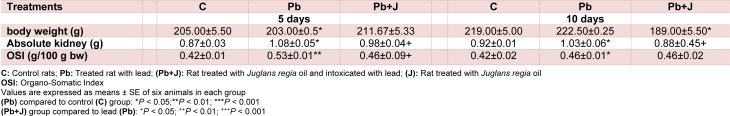
Body, kidney weights of adult rats treated with Pb and/or *Juglans regia* oil for 5 and 10 days

**Table 4:**
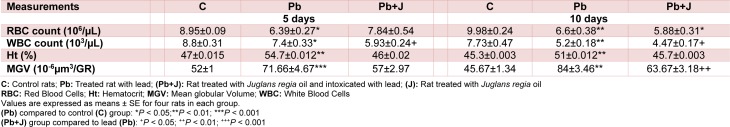
Hematological parameters analysis of adult rats treated with Pb and/or *Juglans regia* oil for 5 and 10 days

**Table 5:**
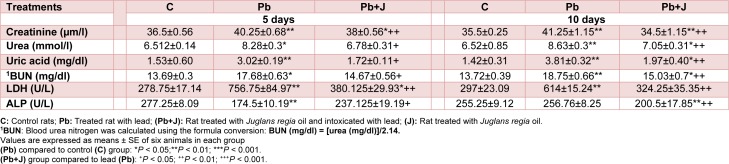
Plasma level of bio-indices of kidney functions in adult rats treated with lead (Pb) and/or *Juglans regia* oil for 5 and 10 days

**Table 6:**
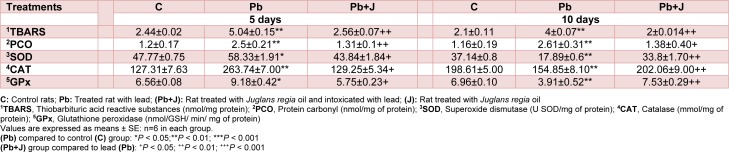
Oxidative status and antioxidant system activity in adult rats treated with lead (Pb) and/or *Juglans regia* oil for 5 and 10 days

**Table 7:**
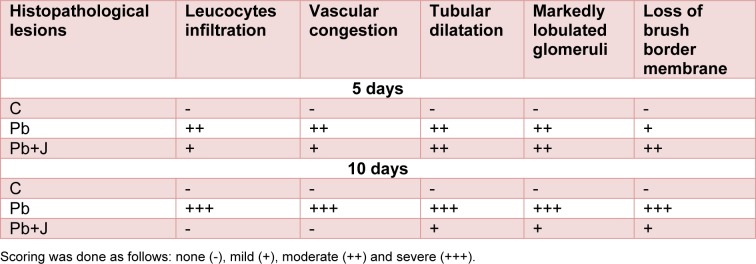
Grading of the histopathological changes in the renal tissues in rat treated just with lead and those treated with lead and *Juglans regia* vegetable oil after 5 and 10 days
